# Trps1 is associated with the multidrug resistance of lung cancer cell by regulating MGMT gene expression

**DOI:** 10.1002/cam4.1421

**Published:** 2018-03-30

**Authors:** Hongxiang Liu, Yi Liao, Meng Tang, Tao Wu, Deli Tan, Shixin Zhang, Haidong Wang

**Affiliations:** ^1^ Cardiothoracic Surgery Department Southwest Hospital Army Medical University (Third Military Medical University) Chongqing China

**Keywords:** Chemotherapy, lung cancer, MGMT, multidrug resistance, Trps1

## Abstract

Multidrug resistance (MDR) often leads to chemotherapy failure of lung cancer and has been linking to the cellular expression of several DNA transcription‐ and repair‐related genes such as Trps1 and MGMT. However, their roles in the formation of MDR are largely unknown. In this study, overexpression/knockdown, luciferase assay and ChIP assay were performed to study the relationship between Trps1 and MGMT, as well as their roles in MDR formation. Our results demonstrated that Trps1 and MGMT expression both increased in drug‐resistant lung cancer cell line (H446/CDDP). Silencing of Trps1 resulted in downregulation of MGMT expression and decrease in the multidrug sensitivity of H446/CDDP cells, while Trps1 overexpression exhibited the opposite effects in H446 cells. Ectopic expression of MGMT had no effect on Trps1 expression, but enhanced the IC50 values of H446 cells or rescued the IC50 values of Trps1‐silenced H446/CDDP cells in treatment of multidrug. Our data further showed that, mechanistically, Trps1 acted as a transcription activator that directly induced MGMT transcription by binding to the MGMT promoter. Taken together, we consider that upregulation of Trps1 induces MGMT transcription contributing to the formation of MDR in lung cancer cells. Our findings proved potential targets for reversing MDR in clinical chemotherapy of lung cancer.

## Introduction

Lung cancer is the first leading cause of cancer‐related deaths in worldwide 1. The high incidences of multidrug resistance (MDR) often result in chemotherapy failure and tumor recurrence of lung cancer 2. Understanding the mechanisms for MDR formation and identifying effective targets to reverse the MDR of lung cancer are critical.

MGMT, also being referred to O6‐alkylguanine‐DNA alkyltransferase (AGAT), can transfer the DNA's O6‐methylguanine adducts or O6‐alkylguanine adducts to its cysteine residues to repair the alkylated damage 3. Studies have reported that suppression of MGMT expression could enhance the treatment efficacy of temozolomide (TMZ) in human melanoma, glioma, and TMZ‐resistant glioma cells 4, 5, 6, 7, 8. Although these studies have indicated the importance of MGMT in the formation of resistance to alkylating agents, there are few reports of the mechanism for regulating the expression of MGMT. Tricho‐rhino‐phalangeal syndrome 1 (Trps1) is implicated in the tricho‐rhino‐phalangeal syndrome (Trps) also known as Langer–Giedion syndrome 9, 10. As an atypical GATA protein, Trps1 plays important roles in development and differentiation in mammals 11, 12, 13, 14, 15. Trps1 also regulated mesenchymal–epithelial transition (MET) during embryonic development 16. Recently, Trps1 was found across the human cancers such as malignant tumor, breast cancer, prostatic carcinoma, and osteosarcoma 17, 18, 19. Therefore, it has been suggested as a potential cytologic tumor marker.

In the present study, we occasionally found that Trps1 and MGMT expressions both increased in cisplatin‐resistant lung cancer cells (H446/CDDP). Therefore, given the transcriptional activity of Trps1, whether Trps1 regulates MGMT expression is quite a significant question for the development of MDR in lung cancer. To elucidate the regulating effect of Trps1 on MGMT expression in lung cancer, we detected the functional interactions between Trps1 and MGMT in a typical small cell lung cancer cell line (H446) by both downregulation and upregulation of Trps1 or MGMT, respectively. We also performed cell viability and IC50 values analysis to evaluate the regulation effect of Trps1 and MGMT on the drug‐resistant ability of lung cancer cells. Moreover, luciferase report systems and ChIP assay were used to further verify the transcriptional activation of Trps1 to MGMT promoter. Our findings elucidated a novel mechanism of Trps1‐MGMT cascade regulated formation of MDR.

## Materials and Methods

### Plasmids

Human Trps1 coding DNA and MGMT coding DNA were cloned into pLenti‐CMV‐GFP‐Puro (Addgene, Cambridge, MA) between BamH I and Sal I sites to form pLenti‐CMV‐Trps1 and pLenti‐CMV‐MGMT vectors, respectively. Trps1 and MGMT coding DNA were amplified by PCR using cDNA prepared from H446 cells; to generate the luciferase reporter vectors, approximately 2.0 kb upstream region from the transcriptional start site of the MGMT gene and three mutant counterparts were cloned into the pGL3 luciferase reporter vector (Promega, Madison, WI). Overlapping PCRs were performed to introduce the mutant sites in MGMT promoters. Then, the promoter fragments were inserted between Xho I and Hind III sites on the pGL3 vector. The oligonucleotide primers used for these constructs are listed in Table 1.

**Table 1:** The oligonucleotide primers used in plasmids construction

Gene	Direction	Primer sequence (5′ to 3′)
Trps1/cDNA	Forward	CGCGGATCCATGGTCCGGAAAAAGAACCC BamH I
	Reverse	ACGCGTCGACTTACTCTTTAGGTTTTCCAT Sal I
MGMT/cDNA	Forward	CGCGGATCCATGCTGGGACAGCCCGCGCC BamH I
	Reverse	ACGCGTCGACTCAGTTTCGGCCAGCAGGCG Sal I
MGMT/Promoter	Forward	CCGCTCGAGTTGTACACACGTAGGGTACG Xho I
	Reverse	CCCAAGCTTTCGGGACGCAAAGCGTTCTA Hind III
MUT1/Overlap	Reverse	CTAGGTTCTGTTTGTATAGTTAATGGAAAGGGGTC
	Forward	GACCCCTTTCCATTAACTATACAAACAGAACCTAG
MUT2/Overlap	Reverse	ATAAGCACCCCAGGGAGTAGATAGATCCCTGGAGGCTTCGG
	Forward	CCGAAGCCTCCAGGGATCTATCTACTCCCTGGGGTGCTTAT

### Cell culture

Human lung cancer cells H446 and H446/CDDP cells were cultured in RPMI‐1640 medium (Corning Cellgro, Herndon, VA) supporting with 10% fetal bovine serum (HyClone, Logan, UT), 100 U/mL penicillin, and 100 mg/mL streptomycin (Corning Cellgro) at 37°C with 5% CO_2_. 1.0 mg/mL puromycin (Sigma‐Aldrich, St. Louis, Missouri, USA) was additionally used to select and maintain the Trps1 and MGMT stably overexpression cells. HEK‐293T was cultured in DMEM (Corning Cellgro) supporting with 10% FBS (HyClone), 100 U/mL penicillin, and 100 mg/mL streptomycin (Corning Cellgro) at 37°C with 5% CO_2_.

### H446/CDDP cells and IC50 detection

Cisplatin‐resistant lung cancer cell line H446/CDDP cells were established by culturing H446 cells with increasing concentration of cisplatin (from 0.5 ug/mL to 1.0 ug/mL) for 3 months. The IC50 was calculated according the cell survival curve which was determined by MTT analysis of living cells after treating with increasing concentration of drug for 72 h.

### Cell viability assay

Cell proliferation was assessed using a MTT based in vitro toxicology assay kit (Sigma‐Aldrich). Briefly, for cells at designed time points, MTT solution (1:10 dilution) was added to and incubated with the cells for 3 h before measuring absorbance at 570 nm.

### Immunofluorescence staining

Monolayer cells were fixed with 4% paraformaldehyde for 15 min at room temperature and permeabilized with 0.2% Triton X‐100 in PBS for 5 min. Cells were then blocked with blocking solution (2 mg/mL BSA in PBS) for 45 min before incubating with anti‐Trps1 rabbit antibody (diluted 1:100, SC‐26976, Santa Cruz Biotechnology, Santa Cruz, CA) or MGMT antibody (diluted 1:300, #2739, Cell Signaling Technology, Inc., Danvers, MA) for 1 h. Then, cells were incubated with a Cy3‐conjugated IgG fraction monoclonal mouse anti‐rabbit IgG (Jackson ImmunoResearch, West Grove, PA) or DyLight 488 AffiniPure Goat Anti‐Rabbit IgG(H+L) (A23220, Abbkine, Wuhan, China) for 1 h at RT. A drop of antifade mount fluid with DAPI (Life Technologies, Waltham, MA) was added to cells for nucleus tinting. Pictures were taken using a NIKON Eclipse E600.

### Establishment of stable cells

Cells overexpressing GFP, Trps1 and MGMT (referred to as Le/GFP, Le/Trps1 and Le/MGMT) were established by lentiviral transduction with each cDNA construct (pLenti‐CMV‐GFP, pLenti‐CMV‐Trps1, or pLenti‐CMV‐ MGMT). In brief, HEK‐293T cells were cotransfected with lentiviral constructs as well as helper vectors pCMV‐VSV‐G plasmid (Addgene) and pCMV‐dR8.2 dvpr plasmid (Addgene). The viral supernatant fraction was collected at 48 h and 72 h after transfection. H446 and H446/CDDP cells were infected with lentiviral particles for 24 h and then selected with puromycin over 1 week. Plasmids were transected using effectene transfection reagent (QIAGEN, Germantown, MD) according to the manufacturer's instructions.

### RNA interference (RNAi)

H446/CDDP cells were grown overnight to approximately 60% confluence and transiently transfected with human Trps1 short interfering RNA (siRNA) siRNA1# 5′ GAGTGATGCTGCAGAACTA3′; siRNA2#5′ GGTCAAGCCAATTGTCAAG3′; or nontargeting siRNA (FITC conjugate)‐A (sc‐36869, Santa Cruz Biotechnology Inc.). H446‐Le/Trps1 cells were transiently transfected with human MGMT short interfering RNA mix (siRNAm) (Genepharma, Pudong, Shanghai, China). Transfection was performed with Lipofectamine RNAi MAX reagent (Invitrogen, Carlsbad, CA) according to the manufacturer's instructions.

### DNA and total RNA isolation, cDNA synthesis, and PCR

Genomic DNA was extracted using Genomic DNA Mini Preparation Kit (Beyotime, Beijing, China) according the instruction. Total RNA was collected using QIAshredder and RNeasy Kit (QIAGEN) according to the manufacturer's instruction. 1.0 ug RNA was reverse‐transcribed using the SuperScript^™^ III Reverse Transcriptase kit (Life Technologies). PCR was performed on the first strand of cDNA with the primers listed in Table 2 using the Phusion reaction system (Thermo Scientific, Waltham, MA) or rTaq (TaKaRa).

**Table 2:** The oligonucleotide primers used in RT and ChIP‐PCR assay

Gene	Direction	Primer sequence (5′ to 3′)
Trps1	Forward	CAATATCTGTGGATATGGT
	Reverse	TGATGTCCTGCAGCACACCAG
MGMT	Forward	GAAATGAAACGCACCACACTGGA
	Reverse	TCAGCCAGGCTGTGCACTGCATCA
MRP	Forward	TTGCCGTCTACGTGACCATTGACG
	Reverse	TTGACAGGCCGTCGCTCGATGCTG
MDR1	Forward	CAGGTTCCAGGATTGGCGTCTTAA
	Reverse	CAGTTGCTATCTTTCCAGCATGCTT
LRP	Forward	CGCTGTGATTGGAAGCACCTA
	Reverse	CGGGAGGCAGCTCTTTCTC
*β*‐actin	Forward	GACGTGGACATCCGCAAAGAC
	Reverse	TCAAGAAAGGGTGTAACGCAAC
Site 1	Forward	TGCAAGTCACAAATACGAAGGTATG
	Reverse	AAGGCCCACATGTTCTAAGCCACC
Site 2	Forward	TGCCAAGGGGTGTGTGACCTCT
	Reverse	CTGTTGTTTTAAGCCACTCAGTGT

### Western blot analysis

Ice‐cold NP‐40 buffer (100 mmol/L Tris, pH 7.4, 80 mmol/L NaCl, 10 mmol/L EDTA, 0.5% Nonidet P‐40, 0.1% SDS) with proteinase inhibitor mixture (Sigma‐Aldrich) was used to extract the protein. The concentration of each protein samples was measured by DC protein assay kit (Bio‐Rad Laboratories, Hercules, CA). 50 ug of protein for each sample was resolved on SDS–PAGE and transferred to nitrocellulose membrane (Invitrogen). The membrane was next incubated overnight at 4°C with primary antibody, followed by incubation with a corresponding horseradish peroxidase‐conjugated secondary antibody (diluted 1:5000; Santa Cruz Biotechnology). Membrane was developed in West Pico SuperSignal chemiluminescent substrate (Pierce). Primary antibodies used were as follows: anti‐MRP (diluted 1:1000, sc‐130065, Santa Cruz Biotechnology, Inc.), anti‐MDR1 (diluted 1:1000, sc‐55510, Santa Cruz Biotechnology, Inc.), antibeta actin (diluted 1:3000, sc‐28287, Santa Cruz Biotechnology, Inc.), anti‐LRP (diluted 1:1000, sc‐23917, Santa Cruz Biotechnology, Inc.), anti‐Trps1 (diluted 1:1000, SC‐26976, Santa Cruz Biotechnology), and anti‐MGMT (diluted 1:1000, #2739, Cell Signaling Technology, Inc.).

### Luciferase assay

Constructs of the MGMT firefly luciferase reporter or their mutants were transfected or cotransfected with the siRNAs, into cells for 48 h. The pRL‐TK Renilla luciferase reporter vector was severed as an internal control (Promega) to normalize firefly luciferase activity. Luciferase activities were measured using the dual‐luciferase reporter assay system (Promega).

### Chromatin immunoprecipitation (ChIP)‐PCR assay

A ChIP assay was performed in H446/CDDP cells using the ChIP assay kit (Beyotime) according to the manufacturer's instructions. Briefly, 10^6^ cells were treated with 1% formaldehyde for 15 min for protein–DNA complexes cross‐linking and sheared with ultrasound pulses on wet ice. Then, chromatin samples were immunoprecipitated with a control IgG or an anti‐TRPS1. PCR was performed using specific primer sets for the Trps1 binding sites in the promoter of the MGMT gene. PCR was performed with 94°C, 1 min; 57°C, 30 s, and 72°C, 30 s for 35 cycles with a final extension for 8 min at 72°C. PCR products were separated on 2% agarose gel and visualized with GelRed (Biotium, Shanghai, China). The PCR primers are listed in Table 2.

## Results

### Increased Trps1 and MGMT expression in drug‐resistant lung cancer cell line

**Figure 1:**
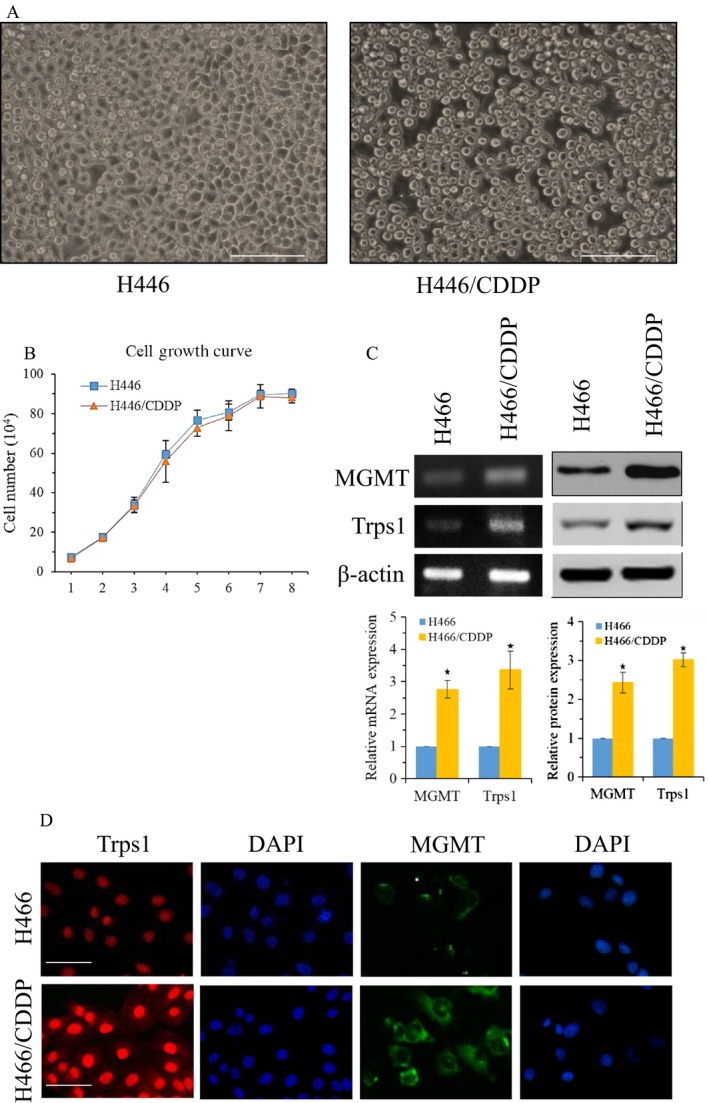
Drug‐resistant lung cancer cells increased expression of Trps1 and MGMT. (A) The morphology of established H446/CDDP cells. Scale bars: 200 mm. (B) Cells growth curve of H446/CDDP and parental H446 cell. (C) Semi‐QPCR and Western blot analyze the expression of Trps1 and MGMT in H446 and H446/CDDP cells. *P < 0.05 versus H446 control; (D) immunofluorescence assay the expression of Trps1 and MGMT in H446 and H446/CDDP cells. Scale bars: 50 mm. All figures represent the average of three sets of independent experiments.

To explore the mechanism of multidrug resistance formation, we first established a cisplatin‐resistant small cell lung cancer cell line H446/CDDP by inducing H446 cells with increasing concentration of cisplatin (from 0.5 ug/mL to 1.0 ug/mL) for 3 months. Comparing to the H446 cells, the H446/CDDP cells became round and diopter degraded (Fig. 1A), while there was no significant change in cell proliferation (Fig. 1B). Multidrug tolerance analysis showed that compared with A549 cells, the IC50 of all tested drugs in H446/CDDP cells was increased, especially with over 20 times resistance against Taxol (Table 3). Furthermore, semi‐QPCR and Western blot analysis revealed the upregulated expression of Trps1 in H446/CDDP cells as well as MGMT (Fig. 1C). By immunofluorescence assay, we further confirmed the increase in Trps1 and MGMT expressions in H446/CDDP cells (Fig. 1D). These results suggest that Trps1 and MGMT might play important roles in the formation of multidrug resistance for lung cancer cells.

**Table 3:** The IC50 of different drugs on H446 and H446/CDDP cells

	CDDP (ug/mL)	EPI (ug/mL)	5‐Fu (ug/mL)	MIT (ug/mL)	Taxol (ug/mL)	VCR (ug/mL)
H446	5.055 ± 0.3	0.12 ± 0.007	260.16 ± 32.5	0.09 ± 0.005	0.007 ± 0.0001	0.03 ± 0.001
H446/CDDP	17.39 ± 0.74	0.63 ± 0.12	1303.96 ± 80.7	0.83 ± 0.09	0.18 ± 0.04	0.31 ± 0.02
Resistance index	3.44	5.25	5.01	9.22	25.71	10.33

### Effects of Trps1 expression on MGMT mRNA and protein levels

**Figure 2:**
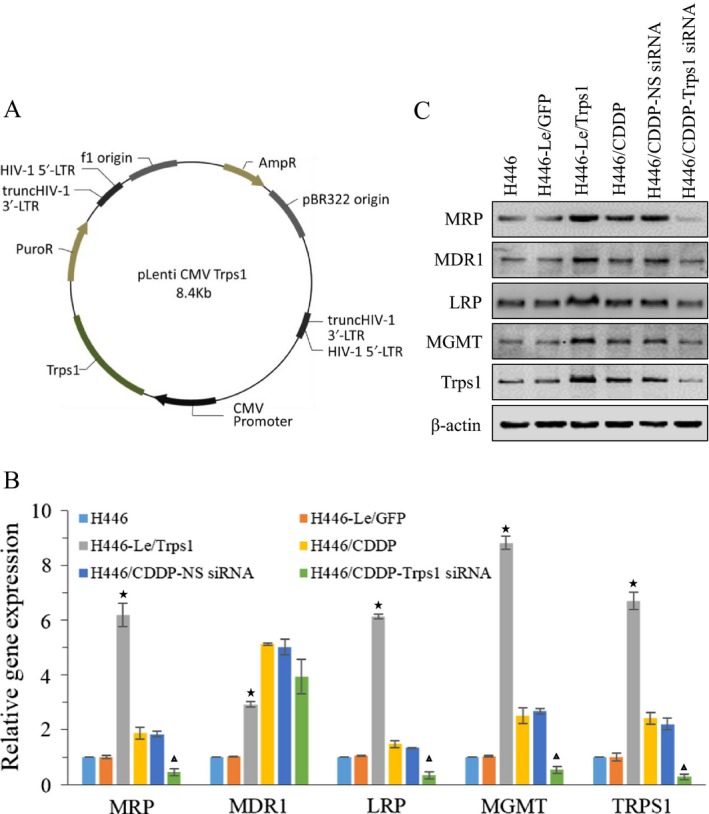
Effects of Trps1 expression on MGMT mRNA and protein levels. (A) The strategy of overexpression Trps1 using plasmid pLenti‐CMV‐GFP‐Puro. (B) QPCR assay the effect of alternation Trps1 expression on the mRNA level of indicated genes. (C) Western blot analyzes the effect of alternation Trps1 expression on the protein level of indicated genes. *P < 0.05 versus H446 and H446‐Le/GFP control groups; ▲, *P* < 0.05 versus H446/CDDP and H446/CDDP‐NS siRNA control groups. All figures represent the average of three sets of independent experiments.

To investigate functional relationships between Trps1 and MGMT, we firstly generated a GFP or Trps1 stably transfected H446 cell lines H446‐Le/GFP or H446‐Le/Trps1. GFP coding sequence was replaced by Trps1 coding sequence, as shown in the map of lentiviral vector (Fig. 2A). Next, we used H446 cell along with H446‐Le/GFP and H446‐Le/Trps1 cells to study the role of trps1 in the regulation of gene expression. Our data showed that the mRNA and protein level of Trps1 gene were increased in H446‐Le/Trps1 cells, and Trps1 overexpression could lead to an increased expression of MGMT as well as MRP, MDR1, and LRP (Fig. 2B and C). In addition, we selected an effective siRNA sequence to knockdown the Trps1 gene expression in H446/CDDP cells (Fig. S1). Our data also demonstrated that silencing Trps1 expression in H446/CDDP cells resulted in decreasing of MGMT expression at mRNA and protein levels, as well as MRP, MDR1, and LRP expressions (Fig. 2B and C). These data indicate that Trps1 could be an upstream mediator of those drug resistance‐related genes.

### Trps1 regulates multidrug sensitivity of lung cancer cells

**Figure 3:**
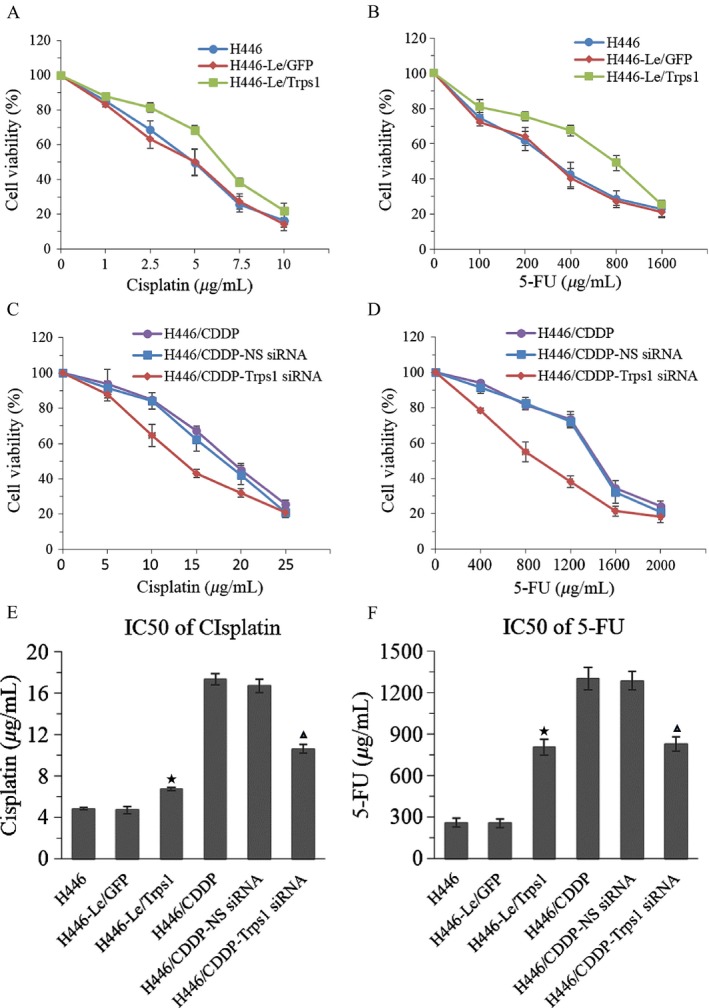
Trps1 regulates multidrug sensitivity of lung cancer cells. (A) The effect overexpression Trps1 on the cell viability of H446 cells after treatment with different concentration of cisplatin. (B) The effect overexpression Trps1 on the cell viability of H446 cells after treatment with different concentration of 5‐FU. C) The effect silencing Trps1 on the cell viability of H446/CDDP cells after treatment with different concentration of cisplatin. (D) The effect silencing Trps1 on the cell viability of H446/CDDP cells after treatment with different concentration of 5‐FU. (E) The IC50 values of cisplatin in Trps1 overexpressed H446 cells or Trps1 silenced H446/CDDP cells. (F) The IC50 values of 5‐FU in Trps1 overexpressed H446 cells or Trps1 silenced H446/CDDP cells. *P < 0.05 versus H446 and H446‐Le/GFP control groups; ▲, *P* < 0.05 versus H446/CDDP and H446/CDDP‐NS siRNA control groups. All figures represent the average of three sets of independent experiments.

Study has reported Trps1 is associated with multidrug resistance of osteosarcoma 20. However, the biological functions of Trps1 in lung cancer have not yet been investigated and reported. Here, we detected the effect of Trps1 on the sensitivity of lung cancer cells to anticancer drugs. The dose–response curves for drug concentrations and cell viability were evaluated by MTT assay. Our data showed a decrease in sensitivity to cisplatin or 5‐Fu in Trps1 overexpressed H446 cells (Fig. 3A and B). On the contrary, Trps1‐silenced H446/CDDP cells presented an increase in sensitivity to cisplatin or 5‐Fu (Fig. 3C and D). The IC50 values of H446‐Le/Trps1 cells were increased in treatment of cisplatin and 5‐Fu, while the IC50 values of H446/CDDP‐Trps1 siRNA cells were significantly decreased (Fig. 3E and F). These results indicate that Trps1 is responsible for MDR of small cell lung cancer.

### Effects of MGMT expression on multidrug sensitivity of lung cancer cells

**Figure 4:**
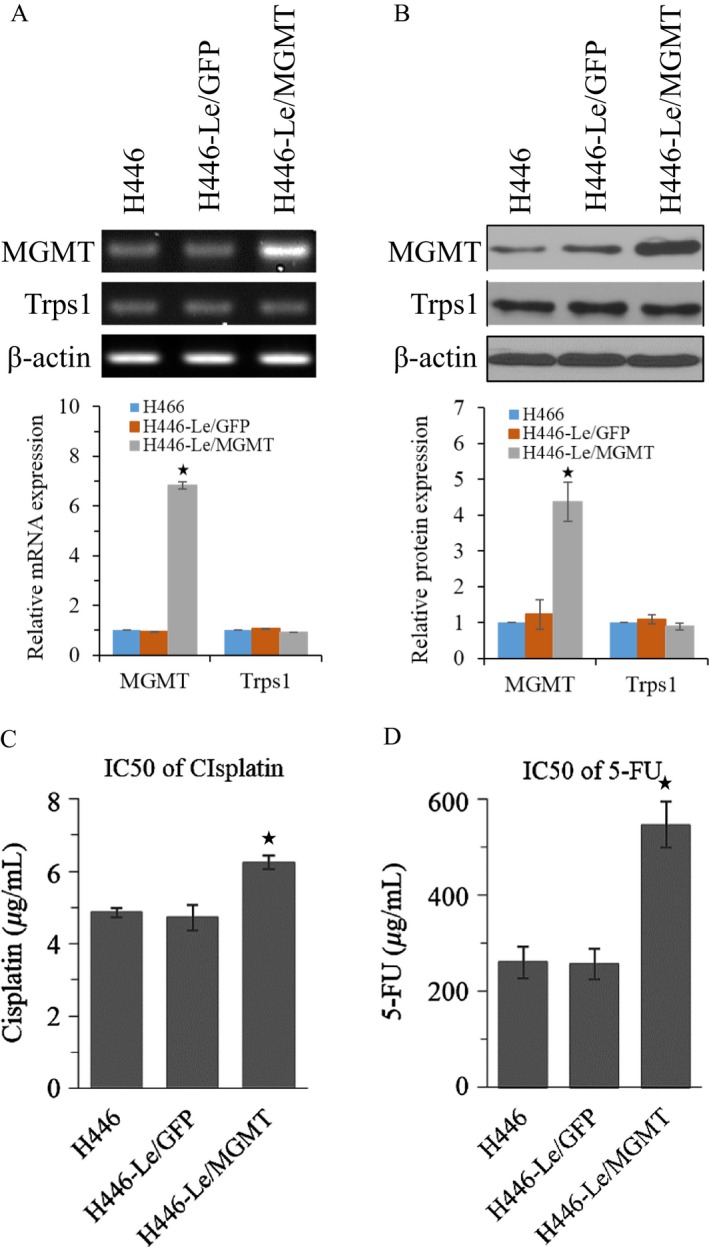
Effects of MGMT expression on multidrug sensitivity of lung cancer cells. MGMT and Trps1 gene expressions were analyzed at mRNA (A) and protein (B) levels in MGMT stably overexpressed H446 cells. *P < 0.05 versus H446 and H446‐Le/GFP control groups; (C) the IC50 value of cisplatin in MGMT overexpressed H446 cells. (D) The IC50 value of 5‐FU in MGMT overexpressed H446 cells. *P < 0.05 versus H446 and H446‐Le/GFP control groups. All figures represent the average of three sets of independent experiments.

**Figure 5:**
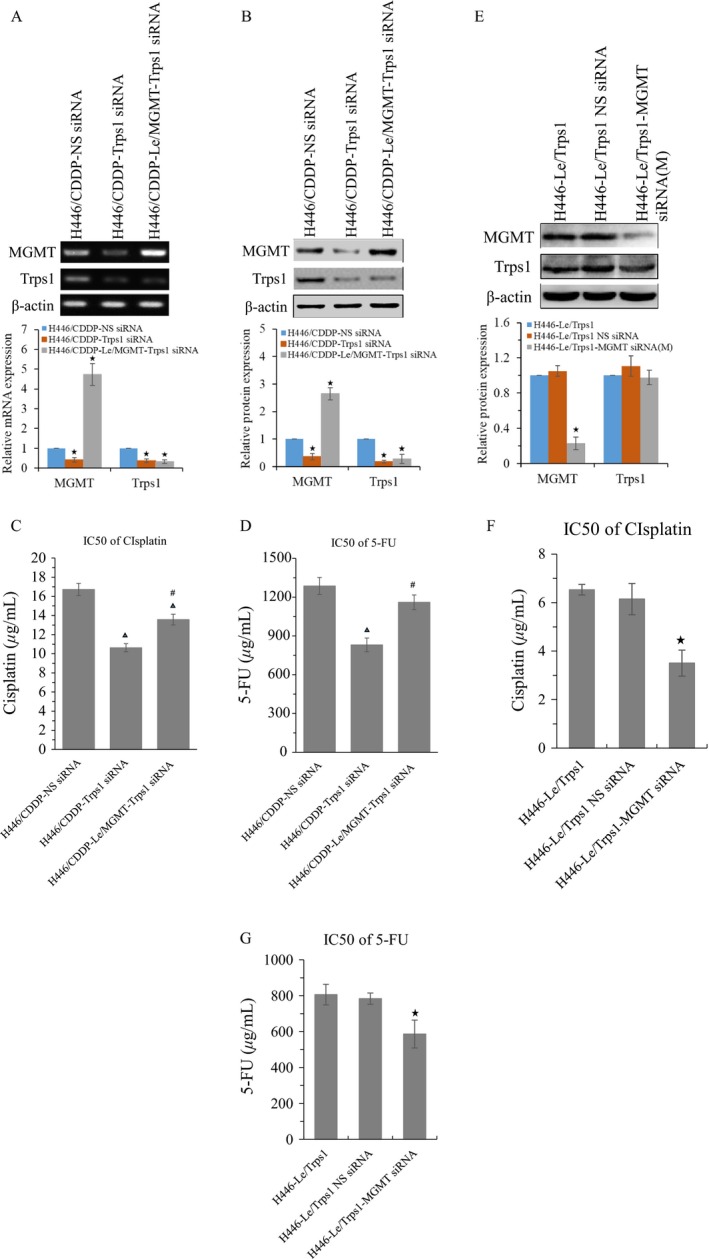
MGMT expression partly reverses the effect of silencing Trps1 on multidrug sensitivity of lung cancer cells. MGMT and Trps1 gene expressions were analyzed at mRNA (A) and protein (B) levels in MGMT stably overexpressed and Trps1 silenced H446/CDDP cells. *P < 0.05 versus H446/CDDP‐NS siRNA control. (C) The IC50 value of cisplatin in MGMT overexpressed and Trps1 silenced H446/CDDP cells. (D) The IC50 value of 5‐FU in MGMT overexpressed and Trps1 silenced H446/CDDP cells. ▲, *P* < 0.05 versus H446/CDDP‐NS siRNA control group; #, *P* < 0.05 versus H446/CDDP‐Trps1 siRNA group. (E) MGMT and Trps1 gene expressions were analyzed at protein level in Trps1 stably overexpressed and MGMT transiently silenced H446 cells. *P < 0.05 versus H446‐Le/Trps1 cells. The IC50 value of cisplatin (F) and 5‐FU (G) in Trps1 overexpressed and MGMT silenced H446 cells. *P < 0.05 versus H446‐Le/Trps1 cells. All figures represent the average of three sets of independent experiments.

To further investigate the functional relationship between Trps1 and MGMT, we established a MGMT stably transfected H446 cell line H446‐Le/MGMT. We found that MGMT overexpression has no effect on the expression of Trps1 gene at neither mRNA nor protein levels (Fig. 4A and B). The IC50 values of H446‐Le/MGMT cells were increased in treatment of cisplatin and 5‐Fu (Fig. 4C and D). In addition, we also established a MGMT stably transfected H446/CDDP cell line H446/CDDP‐Le/MGMT. Semi‐QPCR and Western blot results indicated that this cell line could neutralize Trps1 silencing caused MGMT downregulation (Fig. 5A and B). Our data also showed, for H446/CDDP cells, MGMT overexpression rescued Trps1 silencing caused IC50 decrease in treatment of cisplatin and 5‐Fu (Fig. 5C and D). On the contrary, knockdown of MGMT expression was further preformed to investigate the functional role of MGMT in H446‐Le/Trps1 cells. Our results showed that MGMT siRNA mix could effectively silence the expression of MGMT, while the Trps1 expression was not affected (Fig. 5E). Meanwhile, MGMT silencing could decrease IC50 in treatment of cisplatin and 5‐Fu in H446‐Le/Trps1 cells (Fig. 5F and G). Taken together, our data verify that Trps1 gene is an up mediator of MGMT gene, and Trps1 enhanced antidrug abilities of lung cancer cells are at less partially contributed by MGMT.

### Trps1 induces MGMT transcription through binding to the MGMT promoter

**Figure 6:**
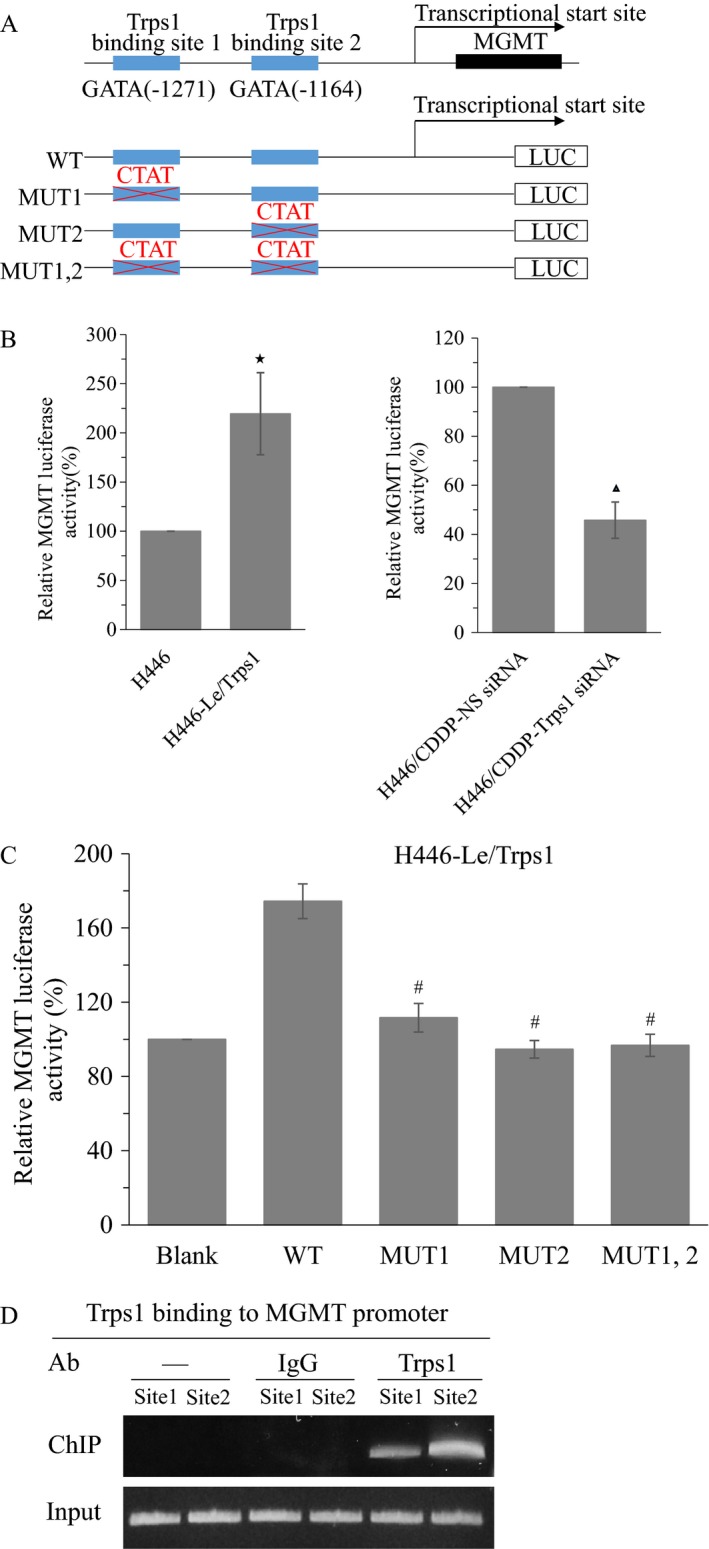
Trps1 transcriptional regulates MGMT expression by binding to the MGMT promoter. (A) Schematic representation of two Trps1 binding sites in the MGMT promoter. (B) H446 cells and H446‐Le/Trps1 cells were transfected with MGMT reporter plasmid, and the luciferase activity was measured. *P < 0.05 versus H446 group. (C) H446/CDDP cells were cotransfected with MGMT reporter plasmid and anti‐TRPS1 siRNA, and the luciferase activity was measured. ▲, *P* < 0.05 versus H446/CDDP‐NS siRNA group. (D) H446‐Le/Trps1 cells were transfected indicated MGMT WT or mutated reporter plasmids, and the luciferase activity was measured. #, *P* < 0.05 versus WT group. (E) ChIP‐PCR assay for the Trps1 binding sites in the promoter of the MGMT gene. All figures represent the average of three sets of independent experiments.

In our study, we have found that overexpression of Trps1 upregulated the mRNA and protein levels of MGMT in H446 cells, while silencing of Trps1 downregulated the mRNA and protein levels of MGMT in H446/CDDP cells (Fig. 2B and C), suggesting that Trps1 increased MGMT transcription. Next, we analyzed the MGMT gene's promoter region using the MATCH (v. 1.0) program (http://www.gene-regulation.com/pub/programs.html) 21. In a region approximately 2.0 kb upstream from the transcriptional start site of the MGMT gene, we found two GATA binding sites for Trps1 (Fig. 6A). Then, a MGMT promoter driving luciferase reporter was constructed to verify that Trps1 directly binds to the MGMT gene promoter to induce MGMT transcription (Fig. 6A). The overexpression of Trps1 increased the MGMT promoter activity in H446‐Le/Trps1 cells (Fig. 6B), and TRPS1 knockdown decreased the promoter activity in H446/CDDP cells (Fig. 6C). Additionally, we constructed three different mutant MGMT promoters to drive the luciferase reporter (Fig. 6A) and measured their promoter activity in H446‐Le/Trps1 cells. Our data showed mutations in either two individual Trps1 binding sites or both blocked the increase in MGMT promoter activity mediated by Trps1 overexpression (Fig. 6D). The binding of Trps1 to the MGMT promoter was further detected by a ChIP assay. Our results demonstrated that unique primers derived from the binding site 1 and binding site 2 showed specific Trps1 binding, while we could not detect any binding by negative or IgG control (Fig. 6E). Collectively, our findings show that Trps1 directly binds to the MGMT gene promoter and induces MGMT transcription in small cell lung cancer cells.

## Discussion

With the most rapidly increasing morbidity and mortality, lung cancer is the leading cause of cancer‐related death in China and is a major public health problem 22. Whether or not surgical resection is operated, chemotherapy is always the major treatment for lung cancer patients. Recently, the development of neoadjuvant chemotherapy has significantly improved the outcomes of early‐stage lung cancer patients 23, 24. However, the development of MDR often leads to the therapeutical failure and tumor recurrence, with only 16% of five‐year survival rate for lung cancer patients 25. Therefore, how to reduce or reverse MDR to improve the effect of chemotherapy is still the emphases for basic and clinical research of lung cancer.

There are four known mechanisms involved in rapid development of cross‐resistance against drugs including enhancement of intracellular drug discharge, inhibition of drug‐induced apoptosis, strengthening of cellular detoxification, and enhancement of DNA damage repair 26, 27. In these processes, cancer cells shift the expression profile including upregulation of transport‐related proteins (P‐gp, MRP, and BCRP), antiapoptosis‐related proteins (p53, Bcl‐2, and survivin), and DNA repair‐related proteins (topological isomerase: Topo I, Topo II; excision repair cross‐complementation group 1: ERCC I and O6‐methylguanine–DNA methyltransferase: MGMT) 28, 29, 30. Studies have demonstrated that overexpression of MGMT resulted in the increased resistance of many anticancer drugs, such as temozolomide, doxorubicin, cisplatin, and etoposide leading to chemotherapeutic failure 28, 31, 32, 33. Although certain inhibitors of MGMT have been developed, the side effects have impeded their application 34, 35. Therefore, to fully elucidate the regulation mechanism of MGMT expression is important to reveal and reversal the MDR formation for lung cancer cells.

In our previous study, we have occasionally found a positive correlation between MGMT and Trps1 in lung cancer cells (data not show). Trps1, a member of the GATA transcription factor family, has been reported to be a transcription suppressor which participates in development and differentiation processes by repressing target genes transcription via specifically binding to “GATA” DNA sequences on their promoters, such as PTHRP, BGLAP, GLI3, and RUNX1 14, 15, 36, 37, 38. While it also has been suggested to be a transcription activator which induces target gene FOXA1 transcription by binding to its promoter in human breast cancer cells 21. By analyzing the MGMT gene's promoter, we have found two GATA binding sites. Therefore, we hypothesized that Trps1 acting as a transcription activator could directly induce MGMT gene transcription by binding to its promoter's GATA binding sites. Our data confirmed our hypothesis and the newly reported role as a transcription repressor of Trps1.

Interestingly, we observed that either overexpression of Trps1 or MGMT in H446 cells could increase the IC50 values of H446 cells in treatment of cisplatin and 5‐Fu, while Trps1 overexpression had a more increasing amount than MGMT overexpression did (Figs. 3 and 4). Meanwhile, our data also showed that overexpression MGMT in Trps1 knockdown H446/CDDP cells only partly rescued Trps1 silencing caused IC50 values decrease (Fig. 5C and D). These data suggested that MGMT was not the only downstream gene regulated by Trps1 to participate in the formation of MDR in lung cancer cells. However, other drug‐resistant‐related downstream targets of Trps1 are still unknown. In our study, we reported that MRP, MDR1, and LRP were also increased in H446/CDDP cells. They are well‐known drug resistance‐related genes. Whether Trps1 participated directly or indirectly in mediating their expression like regulating MGMT gene needs further study. Therefore, it is of great scientific significance to clarify the regulation mechanism and to reveal or even reversal the formation of resistance to chemotherapy drugs for lung cancer cells.

Our data indicated that multidrug resistance of lung cancer cells is partly associated with Trps1‐regulated MGMT gene transcription. These findings suggest that Trps1, as well as MGMT, could be potential targets to reverse MDR in clinical chemotherapy.

## Conflict of Interests

The authors of this article declared they have no conflict of interests.

## Supporting information


**Figure S1.** Selection of effective anti‐Trps1 siRNA. H446/CDDP cells were transfected with NS siRNA, anti‐Trps1 siRNA1# and anti‐Trps1 siRNA 2#, respectively. 48 h after transfection the mRNA (A) and protein (B) levels of Trps1 were analyzed.
