# Supplementary figures and images for: Trps1 is associated with the multidrug resistance of lung cancer cell by regulating MGMT gene expression
Source: Cancer Med. 2018 Mar 30;7(5):1921–32. doi: 10.1002/cam4.1421 (PMC5943538; doi:10.1002/cam4.1421)

A.

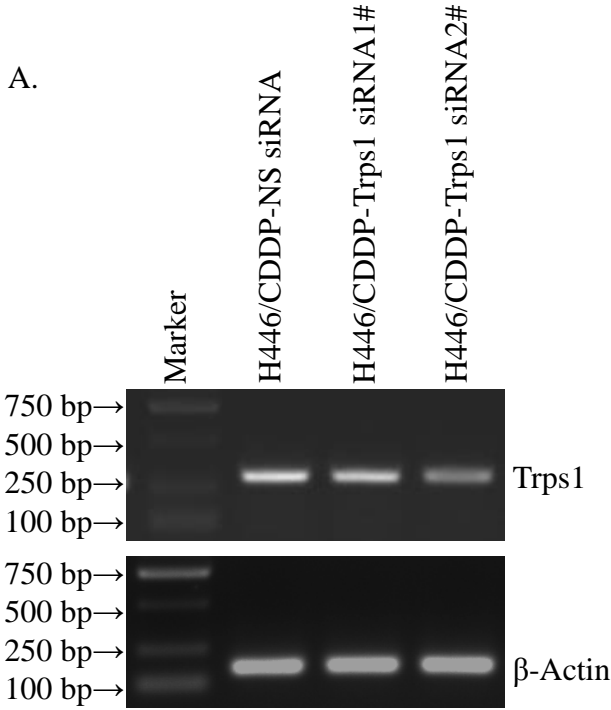

B.

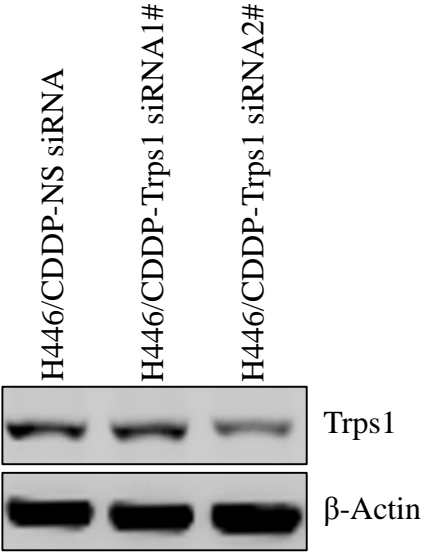

Supplement: Supplementary file 1 — Figure S1. Selection of effective anti‐Trps1 siRNA. H446/CDDP cells were transfected with NS siRNA, anti‐Trps1 siRNA1# and anti‐Trps1 siRNA 2#, respectively. 48 h after transfection the mRNA (A) and protein (B) levels of Trps1 were analyzed. [file CAM4-7-1921-s001.pdf]
